# Adverse Environmental Impact: A Consultant’s Perspective

**DOI:** 10.1100/tsw.2002.350

**Published:** 2002-04-19

**Authors:** Alan W. Wells, Thomas L. Englert

**Affiliations:** Lawler, Matusky & Skelly Engineers LLP, One Blue Hill Plaza, Pearl River, NY 10965, USA

**Keywords:** adverse environmental impact, 316(b) demonstration, Clean Water Act, impact assessment methods, biological models, conditional mortality rate

## Abstract

Environmental consultants are in a unique position to address the practical aspects of a working definition of “adverse environmental impact” (AEI) within Section 316(b) of the Clean Water Act. In our work with the electric utility industry, attorneys, and regulatory agencies, we have encountered numerous and sometimes conflicting interpretations as to what constitutes AEI. In our over 30 years of experience, we have applied most of the approaches suggested for addressing this issue, including biostatistical methods, trend analysis, time series methods, conditional mortality rate models, stock-recruitment models, equivalent adult models, and ecosystem models. In our experience, the paradigm most helpful in bringing about agreement among stakeholders is to (1) create a model of operating scenarios, (2) use empirical data from on-site studies to parameterize the model, (3) convert losses by life stage to equivalent adult losses, (4) convert equivalent adult losses to economic value, and (5) compare scenarios on an economic basis.

### INTRODUCTION

Scientists, engineers, and economists working as environmental consultants are in a unique position to address the practical aspects of a working definition of "adverse environmental impact" (AEI). In our contacts with the electric utility industry, various regulatory agencies, and the legal community, we have encountered numerous and sometimes conflicting interpretations of what constitutes AEI. Over the last 30 or more years, Lawler, Matusky & Skelly Engineers LLP (LMS) has applied most of the suggested approaches with varying degrees of acceptance. In this paper, we share some of our experiences with the hope of providing insight into the development of a useful methodology for meeting the statutory requirements of §316(b) of the Clean Water Act.

To establish a common understanding, we must first ask, "Why do we care about a definition of AEI?" The answer to this requires that we examine the wording of the 316(b) regulation itself.

> Section 316(b) of the Clean Water Act (33 USC §1251 et seq.) requires that: "…the location, design, construction, and capacity of cooling water intake structures reflect the best technology available for minimizing adverse environmental impact."

There are no explicit definitions of AEI in the regulations. Although the U.S. EPA drafted §316(b) regulations in 1976 (40 CFR Parts 401.14, 402.10-402.12 [1976]), those rules were later declared ineffective (Appalachian Power Company v. Train, 566 F.2d 451, 457 [4$^{th}$ Cir. 1977]) and were quickly withdrawn (44 Fed. Reg. 32,956 [June 7, 1979]). In the vacuum of Federal guidelines, two schools of interpretation have evolved around the §316(b) wording.

In our experience, regulatory agencies tend to interpret the wording as "the best technology available for *minimizing* adverse environmental impact" (emphasis added) while the regulated community, guided by their legal advisors, tends to interpret the wording as "the best technology available for minimizing *adverse* environmental impact." The former interpretation stresses that any impact, adverse or otherwise, must be minimized as long as the cost of the remedy is not wholly disproportionate to the benefit achieved (Decision of the General Counsel on Matters of Law Pursuant to 40 CFR Section 125.36(m), No. 63, July 29, 1977, at 23, 27). The latter interpretation effectively establishes a two-tiered hierarchical test (Fig. 1). Only if the impact is deemed "adverse" is the "cost-benefit" analysis necessary.

In practice, what really happens is *not* a hierarchical test. Without a basis for determining AEI, our clients generally elect to submit to the regulatory

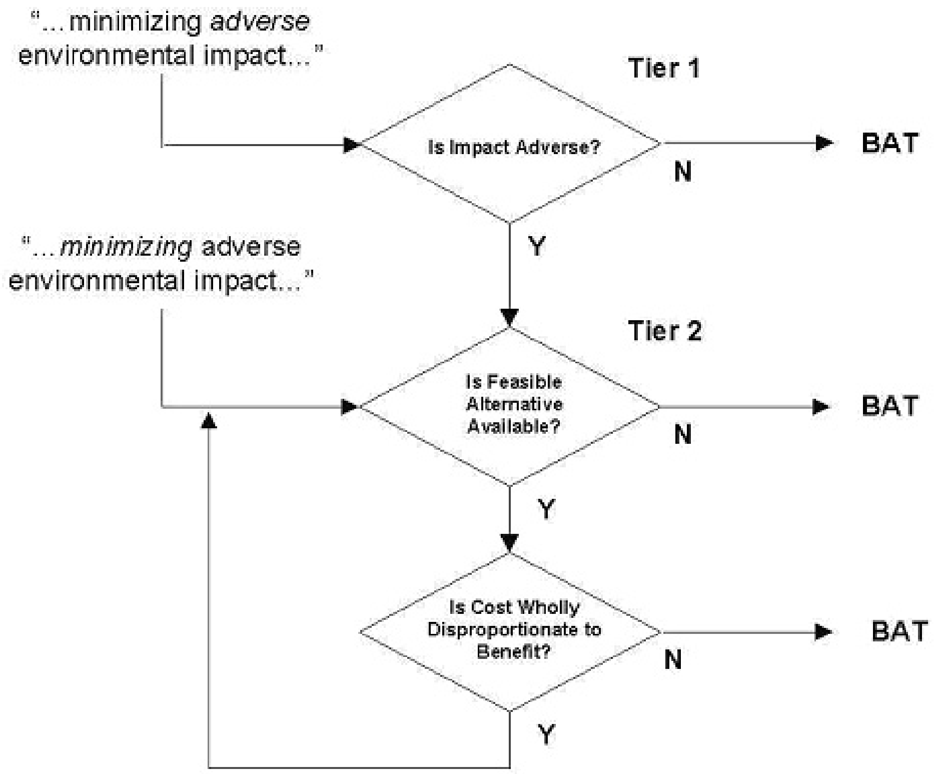

**FIGURE 1.** Interpretative pathways for the phrase "minimizing adverse environmental impact" in Section 316(b) of the Clean Water Act.

agencies a 316(b) demonstration with both elements. First, an assessment of the significance of the impact is made. In the absence of any established guidelines defining AEI, the outcome of the assessment is usually that "no adverse impact" has, or will, occur. But, by the same token, industries requesting the operating permit cannot be assured that their arguments will be accepted, and therefore, they feel compelled to complete the cost-benefit analysis as well.

In the following discussion, we focus on the various approaches LMS has used in the first tier of this process — i.e., the test for AEI — and how effective these approaches were in reaching a mutually agreeable operating permit. As will be seen, we digress periodically to consider aspects of the cost-benefit analysis.

### AEI ASSESSMENT METHODS

Assessment methods fall into three broad categories. Biostatistical Methods generally apply classical statistical techniques to test null hypothesis models. These models are retrospective and cannot be used for *proposed* intakes or for modeling alternative operating scenarios. Predictive Biological Methods typically use intake flow characteristics and population parameters to estimate the near-term and long-term effects of the intake on a single population. Predictive methods can be used in both a retrospective and prospective manner. Community Response Methods attempt to examine the intake effects on the entire biological community (or some part thereof). While community response techniques can be used in a prospective mode, they are more commonly applied in a retrospective mode.

#### Biostatistical Methods

During the initial flurry of 316(b) activity in the mid 1970s through early 1980s, among the first methods to be applied in 316(b) assessments for the determination of AEI were biostatistical methods. These methods fell into two major groups: classical hypothesis testing using analysis of variance (ANOVA) style designs and trend analysis.

ANOVA-style analyses seem to be an outgrowth of the "no prior appreciable harm" model used in 316(a) demonstrations[1] and popularized by a book on environmental sampling by Green[2]. The ideal design was often referred to as a BACI design, meaning before/after/ control/impact. A statistically significant interaction term was taken as evidence of AEI. In cases where the full BACI model could not be used, less persuasive before/after or control/impact comparisons were made. Statistically significant differences between the before and after samples *or* between the control and impacted samples were taken as AEI.

By 1977, papers began to appear that described deficiencies in the ANOVA-style approach[3,4] and our experienced reinforced these warnings. Problems encountered included the following:

- Failure to define the effect size to be detected
- Insufficient statistical power to detect biologically meaningful impacts
- Failure of untransformed data to meet requirements of normality, additivity, independence, and heteroscedasticity
- Failure of transformed data to have real-world meaning
- Interpretation confounded by numerous statistically significant high-order interaction terms

- Inability to use the model in a predictive mode (a critical problem when trying to compare operational alternatives).

During the early stages (early 1980s) of working with the Technical Advisory Group (an advisory group representing numerous regulatory agencies involved in the licensing of the facility) for the Salem Generating Station, in New Jersey, it was very evident that the sampling design necessary for a biostatistical analysis was markedly different from that necessary for other AEI approaches. Given the finite dollar resources available to conduct studies, the TAG readily agreed with the refocusing of sampling priorities to a predictive biological modeling approach (see below) and the abandonment of the biostatistical analysis.

Trend analysis typically requires a relatively lengthy time series of catch-per-unit-effort (CPUE) data. The most commonly used procedure for trend analysis is linear regression. A statistically significant negative slope suggests the potential for AEI. Such an analysis, however, implies a linear population response — a response unlikely for any sustained period of time. A more appropriate but much less often used approach is intervention analysis[5]. Essentially, this analysis is simply the before/after analysis with a longer series of observations and adjustments made for autocorrelated data. We should note that we have rarely encountered data sets of sufficient length (both pre- and postintervention) and consistency to support intervention analysis.

In either the regression or intervention analysis approaches, it is difficult to separate the effects of an intake from other factors, such as overfishing, natural population cycles, or a series of bad year classes due to poor weather. This, in fact, is the major problem with either method. The analysis may describe the overall status of the stock, but it fails to discriminate among causative agents. A good example of the problem is evident in the Hudson River striped bass population. Consultants for the Hudson River Utilities have argued that the population is not significantly affected by the power plants, pointing to increasing stock size documented in CPUE data. Opponents have been quick to point out that the most likely reason for the phenomenal population growth is that the Hudson River striped bass fishery has been closed since 1976 due to PCB contamination (e.g., [6]).

### Predictive Biological Methods

Predictive biological models fall into three general categories, sometimes referred to as: Absolute Damage Models, Relative Damage Models, and Long-Term Damage Models. As one progresses from absolute, to relative, to long term, the number of input parameters, sampling program cost, and relevance to

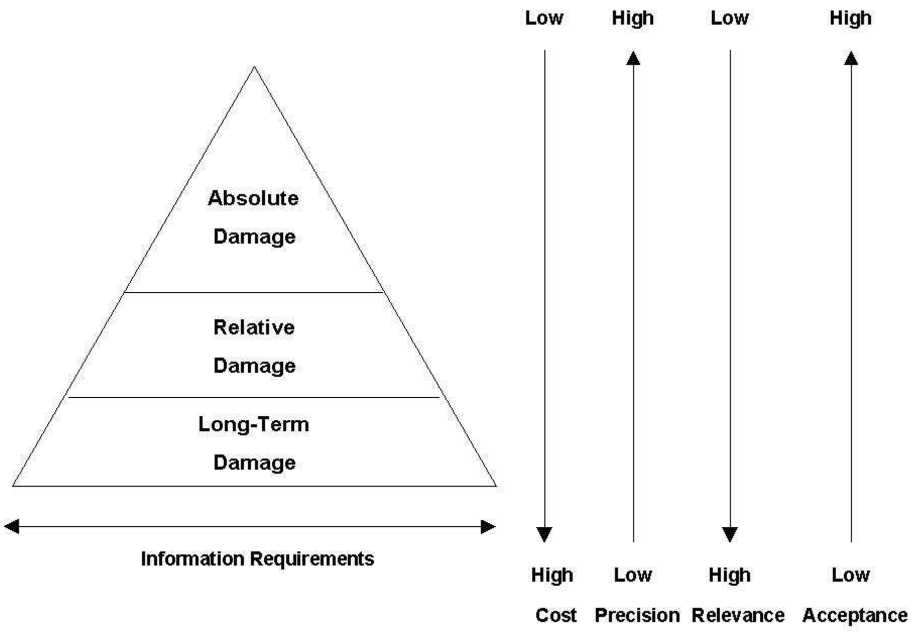

**FIGURE 2.** Relationships among various Section 316(b) damage assessment modes.

AEI determination increase. With each new input parameter, however, overall relative model precision tends to decrease and the potential for acceptance by regulatory agencies decreases (Fig. 2).

Absolute damage models yield the estimated number of organisms killed by entrainment and impingement. These losses generally are presented as number by life stage, but, we have found that expressing losses as equivalent adults[7,8] is more helpful in understanding the biological significance of the losses and in assessing the economic value:

$$N_A = \sum_{i=1}^{n} N_i S_i$$

where n = number of life stages, $N_i$ = number of life stage *i* killed, and $S_i$ = survival probability from life stage *i* to adulthood.

Losses expressed in this manner are more readily compared to other known sources of mortality, particularly to a commercial or recreational fishery. It is generally a relatively easy matter to convert numerical losses expressed in this manner to standing stock biomass (usually pounds), production, or economic value. We have not had regulatory agencies object to this form of analysis, and in several instances, they have specifically requested this approach.

In general, the absolute damage model approach requires only routine on-site data collection. An additional advantage of this approach is that the derived concentrations of entrained or impinged organisms in the intake water can be used to compare different operating scenarios. One need only alter the intake flow, through-plant exposure temperature and exposure duration, or impingement mortality rate. Operating alternatives can then be compared directly on the basis of numeric loss, biomass, or dollars.

Clearly, basing an AEI assessment on absolute losses alone can be misleading. For example, large numeric losses are potentially more harmful to a small source population than to a large population. Relative damage models attempt to estimate the loss as a fraction of the source population. They are sometimes referred to as fractional loss models or conditional mortality rate (CMR) models. (The conditional mortality rate is the fraction of the year class lost in the absence of all other sources of mortality).

One of the first attempts at estimating “relative damage" was through the use of hydrodynamic models[9]. These models are based on the assumption that the early life stages of fish are transported by the hydrodynamics of the source waterbody. Hydrodynamic-based models were hotly contested during the early days of the Hudson River hearings[10,11], primarily because of the inability to adequately capture fish behavior. It was soon realized that even the very young larvae of many species do not behave strictly as passive particles, but instead have developed behavioral mechanisms to adapt to their particular environment. Hydrodynamic models soon gave way to more empirically based models such as the Empirical Transport Model[12,13] and the Empirical Impingement Model[14]. In our experience, the use of these empirically based models is generally well accepted by regulatory agencies and, in many cases, are explicitly required as part of the permitting process. Empirical models still have a number of limitations, however. Among them are:

- CMR values are relative to an arbitrarily defined study area.
- The models often unrealistically assume a closed system (modifications to relax this assumption are available).
- Confidence intervals are difficult to establish.
- Models such as the Empirical Impingement Model require estimates of population size and mortality rates, both of which are difficult to obtain with precision.
- Models do not include compensatory or depensatory processes.

The primary problem with "relative damage" models, however, is the translation of the CMR to AEI. Once the CMR is known, is it adverse? We have frequently run across a “rule of thumb” stating that a CMR value greater than

10% is adverse (15% for forage species) or has the potential for adverse impacts (e.g., [15]). On occasion we have also encountered agencies that consider CMRs of less than 1% adverse. There is, however, no firm biological foundation for either of these thresholds. The "adversity" of the value is highly dependent on the circumstance and no general guideline seems possible.

The only practical way to assess the consequences of a given CMR on a population is to incorporate the value into a "long-term damage" model. Among the first of these models to be used was the Leslie Matrix population projection model. We found this approach to be of little help in the assessment process and not well received by guidance committees. Without the incorporation of density-dependent mechanisms, even the slightest loss drives the modeled population to eventual extinction. Stock-recruitment models based on Ricker, Beverton-Holt, or similar functions[16,17] are somewhat more useful, but they were poorly suited to nonequilibrium populations or iteroparous spawners[18]. The stock-recruitment approach met with considerable resistance during the initial Hudson River hearings in the 1970s, primarily because of the difficulty in estimating the model parameter[19,20]. In our recent experience, RAMAS[21] and approaches derived from Hilborn and Walters[22] seem to have gained some acceptance. These models combine population projection matrices with flexible density-dependent recruitment models. Bayesian maximum likelihood methods[23] coupled with meta-analysis[24] have made important gains in obtaining better estimates of the critical density-independent response parameters. Individual based models, such as Rose et al.[25], can also be useful in overcoming limitations of estimating density-dependent parameters.

"Long-term" damage models allow us to examine the effects of various CMR values on a population over time. Additionally, by incorporating Monte Carlo techniques, we can use the models to assess the probability of certain outcomes. But this increased usefulness comes at a price—increased data requirements. Information on age-specific growth, mortality and fecundity values, year (and sometimes age) specific fishing rates, carrying capacity ($K$) and the intrinsic rate of population increase ($r$) are needed. It is also being widely recognized that $K$ and $r$ can change in response to density-dependent and density-independent factors[26,27].

We have encountered widely varying responses to the inclusion of "long-term" damage models. In the Hudson River hearings during the 1970s, much of the nearly 10-year-long debate was spent arguing over these types of models — in particular, the degree of density-dependent compensation. In the long run, these models contributed little to the final outcome. We should point out that a consensus was reached on CMR values and that they did, in fact, play an important role in settling the case[28]. In at least one major case in which we were involved during the early 1980s after the Hudson River case, the technical advisory body explicitly cautioned us not to use "long-term" models. The advisors did not want to repeat the Hudson River turmoil, and they believed they could determine AEI without this information.

In several more recent cases, opposition to "long-term" models seemed to have waned[29,30]. In one instance, while we were not discouraged from using the approach, the reviewers ignored the results in reaching their final recommendations. In another ongoing case, the technical advisory body actively encouraged use of these models, but as the case progresses, it is becoming increasingly unclear how important this technique will ultimately be.

A "long-term" damage assessment is a complex undertaking and close cooperation with resource agencies seems to be required for the effort to be credible. Such a close relationship, however, can lead to several difficulties. One is a gradual change in scope and focus. In a recent application, we worked closely with the utility, resource agencies, and other consultants, all with varying degrees of understanding of the modeling process. Each model input parameter was thoroughly discussed and agreed upon before incorporation into the model. As understanding of the model grew, various members would suggest changes or additional output. While some changes were easily incorporated, others would have required fundamental changes to the model's underlying structure — changes that would have required considerable reprogramming and resulted in serious time delays. Without the changes, the proposing committee member(s) began to lose confidence in the unmodified model.

Despite the enhanced insight offered by "long-term" damage models, we are still saddled with the fundamental problem of interpretation of AEI. Is there a biological benchmark that can be considered adverse? One frequently suggested benchmark — stemming from the 1975 Draft Guidance Manual[31] — is a reduction in maximum sustainable yield (MYS) or optimum sustainable yield (OSY). Unfortunately, given any power plant CMR and realistic values of density-dependent response, "long-term" damage models will *always* show a reduction in MSY or OSY. Consequently, any loss, no matter how small, would be considered as adverse. Fisheries benchmarks — such as $F_{MSY}$ $F_{OSY}$, or several other related measures — emphasize the fact that intake losses are clearly part of the larger picture of fisheries resource allocation. The loss of aquatic organisms can be allocated among all user groups, including commercial and recreational fishermen. This larger perspective, however, needs to be addressed by the resource agencies, not by the individual 316(b) applicant. It should also be noted that fisheries benchmarks are workable for species supporting commercial or recreational fisheries, but do not appear to be applicable to nonexploited species due to lack of data availability.

### Community Response Methods

While most of the analyses we have conducted have been at the population level, it is also worthwhile to examine impacts from a community perspective. Ideally, populations selected for study under 316(b) represent a cross-section of the biological community that occupies the source waterbody. Population-level results can then be generalized to the community level. We have found that, in practice, "target species" disproportionately represent commercially or recreationally important species or reflect an advisory group's or group member's special interest in a particular species.

A number of techniques have been devised for looking at community-level effects. Among them are such simple methods as species richness, diversity, evenness, and percent similarity, and more complex forms such as indices of biotic integrity, loop analysis, and ecosystem models. We have found the simple forms to be useful in some circumstances. For example, in one recent application, richness, diversity, and evenness measures demonstrated an obvious change in fish community structure[30]. An examination of assemblages within the study community (e.g., benthic vs. pelagic, resident vs. migrant, and northern vs. southern origin) demonstrated nearly identical population trends among all groups. From this knowledge, we were able to narrow down the list of causative agents. We have had little call for the more complex community approaches; particularly loop analysis and ecosystem models. These methods are not readily understood by reviewers and generally require impractical amounts of data.

One of the primary difficulties with the application of a community-level approach is the inevitable blurring of the lines between 316(b) (with its AEI test for intake structures) and 316(a) (with its "protection of a balanced indigenous community" test for discharges). In the past, we have encountered several agencies that have required a clear distinction between 316(a) and 316(b). While intake and discharge effects certainly are interdependent, current statutes require that they be kept distinct. We generally conduct community analyses, such as described above, under 316(a).

## DISCUSSION AND CONCLUSIONS

In our over 30 years of corporate experience, we have found that it is generally the simple approaches, in combination, that work best for addressing Section 316(b) issues. In particular, we have found:

- Absolute damage estimates are always required and are a necessary first step to any other analysis.

- Relative damage estimates are frequently requested by agencies, but they can be relatively less precise than absolute damage estimates and are seldom definitive in AEI assessment. There is no practical AEI benchmark because all situations are different.
- "Long-term" damage estimates may provide useful insights into the relative importance of various sources of impact and into the probability of outcomes of various actions. However, these methods, relative to the "absolute" and "relative" methods, require data on an increased number of parameters, some of which are difficult to estimate with precision. Uncertainty in the model input parameters leads to lack of confidence in the results. In the past, we have found "long-term" damage assessment models even less influential than "relative" damage assessments in 316(b) negotiations.
- Simple "community" level analyses can be important, but are best left to 316(a) demonstrations. This is not to say that community level analyses should not be conducted; certainly there are good ecological reasons for examining community responses. Under the current statutes, however, the analysis is more appropriately placed in context of 316(a) (i.e., maintenance of a balanced indigenous community). If both 316(a) and 316(b) Demonstrations are submitted simultaneously, as is often the case, the community analysis can be easily referenced from the 316(b) portion.

The most effective approach we have found can be broken down into the following steps:

1. Construct a simple model of plant operations that incorporates alternative operating modes.
2. Using the empirically observed concentrations of organisms, compute "absolute" losses by life stage (or age class) for the existing operational mode and for several alternative scenarios.
3. Use the equivalent adult model to translate losses by life-stage to losses of adults.
4. Convert the adult losses to dollars. (Conversion to economic value is not without problems and controversy. In some cases the American Fisheries Society monetary values for fishes have been used[32] or, more frequently, the dockside value for commercially important species. Maryland, under Natural Resources Article Section 8-1405(c) incorporates economic loss values directly into their statute. None of these methods capture the all of the indirect environmental and social values of the resource. Methods attempting to incorporate these measures are infrequently encountered.)
5. Compare alternative operating modes on a dollar value basis.

The preceding approach requires little more than an understanding of the plant operation, routine on-site monitoring data, an economic value for organism losses, and a few basic life-history parameters. What the approach does *not* require is a definition of "adverse." Our paradigm shifts the focus from one of biology to one of economics. Ultimately, we must recognize that "adverse" has no true biological meaning. It is only from a human and societal perspective that can we say what is adverse. As scientists and engineers, we can contribute to the understanding of various alternatives by expressing the consequences of alternative actions in terms that stakeholders understand.
